# The Association between the Epicardial Adipose Tissue Thickness and Oxidative Stress Parameters in Isolated Metabolic Syndrome Patients: A Multimarker Approach

**DOI:** 10.1155/2014/954045

**Published:** 2014-11-02

**Authors:** Bulent Demir, Esra Demir, Gonul Acıksarı, Turgut Uygun, Irem Kırac Utku, Asuman Gedikbasi, Ilker Murat Caglar, Osman Pirhan, Hande Oktay Tureli, Ersan Oflar, İsmail Ungan, Serkan Ciftci, Osman Karakaya

**Affiliations:** ^1^Department of Cardiology, Bakırkoy Dr. Sadi Konuk Education and Research Hospital, Tevfik Sağlam Caddesi No. 11, Zuhuratbaba Mahallesi, Bakırköy, 34156 Istanbul, Turkey; ^2^Department of İnternal Medicine, Bakırkoy Dr. Sadi Konuk Education and Research Hospital, Turkey; ^3^Department of Cardiology, İstinye State Hospital, Turkey; ^4^Department of Biochemistry, Bakırkoy Dr. Sadi Konuk Education and Research Hospital, Turkey

## Abstract

The risk for cardiovascular diseases and type 2 diabetes mellitus significantly increases in the patient population with metabolic syndrome (MeS). The present study aimed to investigate the association between the epicardial adipose tissue thickness (EATT) and the oxidative stress parameters in MeS patients. The study included 181 patients as a patient group of 92 consecutive patients with MeS and a control group of 89 consecutive patients with similar age and gender. EATT was evaluated by transthoracic echocardiography. Serum levels of total oxidant status (TOS), total antioxidative capacity (TAS), paraoxonase-1 (PON-1), and arylesterase activities were measured. EATT was higher in the MeS group compared to the control group (6.0 ± 2.0 mm and 4.0 ± 1.0 mm, resp.; *P* < 0.001). The level of TOS was higher in the MeS group compared to the control group (*P* < 0.001). Additionally, the TAS level was higher in the MeS group compared to the control group (*P* < 0.001). Furthermore, the serum levels of PON-1 and arylesterase were lower in the MeS group compared to the control group (*P* < 0.001). EAT may cause an increased risk of cardiovascular diseases by leading to increased oxidative stress in patients with MeS.

## 1. Introduction

Metabolic syndrome (MeS) is defined as a cluster of risk factors involving abdominal obesity, impaired fasting glucose, hypertension, and dyslipidemia [1]. The risk for developing type 2 diabetes mellitus and coronary artery disease is significantly increased in MeS patients relative to the normal population [2]. Therefore, the treatment of the patients with MeS, which is currently increasing in prevalence, and the prevention of developing MeS are highly important for the public health.

Abdominal obesity, namely, visceral adipose tissue, has an important role in the pathogenesis of the MeS [2]. In recent years, visceral adipose tissue has been shown to play a role in the pathogenesis of cardiometabolic diseases, such as MeS, diabetes mellitus, and coronary artery disease, by producing several cytokines and hormones called adipokine [3, 4]. In other words, visceral adipose tissue is metabolically active adipose tissue. Epicardial adipose tissue (EAT) is a newly defined adipose tissue, which is considered a component of visceral adipose tissue. In recent clinical trials, a significant association has been established between the epicardial adipose tissue and MeS [5, 6].

Increased oxidative stress has been shown to have an important role in the pathogenesis of MeS, just as in many cardiometabolic diseases [7]. In particular, the impaired balance between the prooxidant/antioxidant systems in favor of prooxidant system results in low-grade inflammation, endothelial dysfunction, and insulin resistance [8]. Hopps et al. defined oxidative stress as a new component of MeS [7]. The increased expression of Rac1 protein, which has a key role in oxidative stress regulation, has been shown to play a role in the increased oxidative stress associated with obesity [9]. Therefore, visceral obesity leads to the development of MeS by also inducing the increase in oxidative stress. In this regard, epicardial adipose tissue, which is a component of visceral adipose tissue, may also cause an increase in oxidative stress. It has been shown that epicardial adipose tissue has more oxidative stress markers than the subcutaneous adipose tissue in a study conducted with proteomic analysis [10]. Accordingly, the epicardial adipose tissue may play a role in the development of MeS by causing oxidative stress.

To the best of the researchers' knowledge, although there are no sufficient data that demonstrate the association of epicardial adipose tissue with MeS in the literature, there are no sufficient data discussing the relation between the epicardial adipose tissue and oxidative stress. The present study aimed to investigate the association among the epicardial adipose tissue and total oxidant status (TOS), total antioxidant status (TAS), oxidative stress index (OSI) from the oxidative stress markers and paraoxanase-1 (PON-1), and arylesterase (ARE) from the antioxidant enzymes.

## 2. Materials and Methods

The present study was designed as a prospective, cross-sectional study. The study included 92 consecutive patients who were admitted to the Cardiology and Internal Diseases Polyclinic and were diagnosed with MeS between December 2012 and January 2014. Eighty-nine patients who were of similar age and demographics and who were excluded from the MeS diagnosis were included in the control group.

*The exclusion criteria* were as follows: diabetes mellitus or the use of oral antidiabetics, coronary artery disease, congestive heart failure, atrial fibrillation, congenital heart disease, valvular heart disease, myocarditis, pericarditis, cardiomyopathy, impaired renal functions (creatinine > 1.5 mg/dL), neoplasia, autoimmune disease, chronic inflammatory disease, active infection, chronic hepatic disease, and antioxidant vitamins or medications.

For each patient, a detailed cardiovascular medical history was recorded and a detailed physical examination was performed. The waist circumference of the patients was measured parallel to the floor from the narrowest distance between the lowest costa and processus spinosus as the patients were standing and the belly was opened. The body mass index was calculated using the weight (kg)/height (m^2^) formula. Additionally, the patients included in the study were assessed by twelve-channel electrocardiography. Systolic and diastolic arterial blood pressure levels were measured from both arms of patients after a rest period of 20 minutes using a mercury manometer.

Hypertension was considered as systolic blood pressure ≥ 140 mmHg at a minimum of three different measurements when the patient was at rest; a diastolic blood pressure ≥ 90 mmHg; or receiving antihypertensive medications. Diabetes mellitus was defined as a fasting blood glucose ≥ 126 mg/dL or still receiving antidiabetic treatment. The patients with a fasting plasma glucose level from 100 to 126 mg/dL were considered to have impaired fasting glucose. Hyperlipidemia was defined as total cholesterol ≥ 200 mg/dL or triglyceride levels > 150 mg/dL [11]. Regardless of the amount, the patients who were actively smoking were considered as smokers.

Metabolic syndrome was diagnosed based on the International Diabetes Federation (IDF) Criteria 2005 [12]. Metabolic syndrome was defined as having at least two of the following criteria in addition to the abdominal obesity criterion that was defined as a waist circumference > 94 cm in men and >88 cm in women: (1) elevated levels of triglycerides: ≥150 mg/dL (1.7 *μ*mol/L); (2) reduced levels of HDL cholesterol: <40 mg/dL (1.03 *μ*mol/L) in men and 50 mg/dL (1.29 *μ*mol/L) in women; (3) elevated blood pressure: systolic blood pressure ≥ 130 mmHg or diastolic blood pressure ≥ 85 mmHg; (4) elevated fasting plasma glucose: elevated fasting plasma glucose ≥ 100 mg/dL (5.6 *μ*mol/L).

### 2.1. Measuring Epicardial Adipose Tissue Thickness with Echocardiography

All patients included in the study had a detailed two-dimensional, M-mode, and Doppler echocardiographic assessment by two experienced echocardiographers who were blind to the biochemical data. A Vivid S-5 (GE Vingmed, Horten, Norway) was used as the echocardiography device with a 2.5–3.5 MHz probe. Epicardial adipose tissue is defined as the adipose tissue between the visceral pericardium and the outer margin of the myocardium [13]. It is viewed as the relatively echo-free area between the outer margin of the myocardium and the visceral layer of the pericardium on echocardiography. In the present study, the epicardial adipose tissue thickness was recorded by measuring at the end-diastole in the way that the right ventricle was perpendicular to the free wall on the parasternal longitudinal axis and transverse images, in accordance with the literature [13, 14]. The aortic annulus and interventricular septum were used as the anatomic markers in order to establish the perpendicularity of the right ventricle to the free wall [13]. To minimize the margin of error, the average of the measurements made similarly in five consecutive cycles was determined as the final epicardial adipose thickness.

### 2.2. Laboratory Parameters

#### 2.2.1. Blood Sample Collection

Venous blood samples were collected in tubes from the antecubital vein, followed by overnight fasting. The tubes were centrifuged at 4000 rpm (10 min) to remove the plasma and serum. The plasma and serum samples were kept at −80°C until analysis of PON 1 activity and TAS and TOS is done.

#### 2.2.2. Measurement of Paraoxanase-1 and Arylesterase Activity

Paraoxonase and arylesterase activities were determined using a novel automated measurement method developed by Erel (Rel Assay, Turkey) [15]. Briefly, the rate of paraoxon hydrolysis was measured by the increased absorbance at 412 nm at 25°C. The PON activity is expressed as U/L serum. The coefficient of variation (CV) for individual samples was 1.8%. Arylesterase activity was measured spectrophotometrically using phenyl acetate. The reaction was started by the addition of the serum; the increase in absorbance was read at 270 nm. Enzymatic activity was calculated from the molar absorptivity coefficient of the produced phenol. One unit of arylesterase activity was defined as 1 *μ*mol phenol generated/min under the defined assay conditions and expressed as U/L serum. The CV for individual serum samples was 3.3%.

#### 2.2.3. Measurement of the Total Antioxidative Capacity (TAS)

The total antioxidant status (TAS) of the plasma was determined using a novel automated measurement method developed by Erel [16]. In this method, hydroxyl radical, which is the most potent biological radical, is produced. The assay measures the antioxidative effect of the sample against potent free radical reactions that are initiated by the hydroxyl radical produced. The precision of the assay is excellent and is lower than 3%. The results are expressed as *μ*mol Trolox equivalent/L.

#### 2.2.4. Measurement of the Total Oxidant Status (TOS)

The TOS of serum was determined using a novel automated measurement method, also developed by Erel [17]. Oxidants present in the sample oxidize the ferrous ion-o-dianisidine complex to ferric ions. The oxidation reaction is enhanced by glycerol molecules, which are abundant in the reaction medium. The ferric ion generates a colored complex with xylenol orange in an acidic medium. Color intensity, which can be measured spectrophotometrically, is related to the quantity of oxidant molecules present in the sample. The assay is calibrated with hydrogen peroxide and the results are expressed in terms of micromolar hydrogen peroxide equivalents per liter (*μ*mol H_2_O_2_ equiv./L).

#### 2.2.5. Oxidative Stress Index

The OSI is defined as the ratio of the TOS to TAS level, expressed as a percentage. For the calculation, TAS units were changed to *μ*mol/L and the OSI value is calculated according to the following formula: OSI (arbitrary unit) = TOS (*μ*mol H_2_O_2_ equiv./L)/TAS (*μ*mol Trolox equiv./L).

#### 2.2.6. Other Variables

*Serum hsCRP levels* were measured by nephelometry using BN II nephelometer (Siemens Healthcare Diagnostics, USA). Serum glucose, urea, creatinine, LDL cholesterol, HDL cholesterol, triglyceride, TSH, fT3, fT4, and other biochemical parameters were determined by an Abbott Architect C16200 Integrated System and using commercial kits (Abbott Laboratories, IL, USA). Serum insulin was determined by an Immulite 2000 chemiluminescence autoanalyzer and using commercial kits (Siemens Healthcare Diagnostics, USA). Insulin resistance was estimated with homeostasis model assessment-insulin resistance index (HOMA-IR) [18]. HOMA-IR was calculated with the following formula: fasting plasma glucose (mg/dL) × fasting serum insulin (mU/L)/405. Complete blood count was determined in a Coulter LH 750 autoanalyzer (Beckman Coulter, CA, USA).

### 2.3. Statistical Analysis

Data were analyzed using SPSS 22.0 for Windows software (SPSS Inc., Chicago, IL, USA). Frequency, ratio, mean, minimum, maximum, and standard deviation values were used in the descriptive statistics. The Kolmogorov-Smirnov test was used to control the data distribution. The independent samples* t*-test and Mann-Whitney* U*-test were used to analyze quantitative variables. The chi-square test was used to analyze qualitative variables. Spearman's correlation tests were used for the correlation analysis. The linear logistic regression analysis was performed to determine the level of effect on the parameters. Standard beta coefficients and 95% confidence intervals (CI) were calculated. The receiver operating characteristics (ROC) curve analysis was used to calculate the required cut-off values to distinguish MeS patients with maximum sensitivity and specificity. *P* values < 0.05 were considered statistically significant.

## 3. Results

The patient demographics and clinical and laboratory characteristics are summarized in Table 1. There was no statistically significant difference in age, gender, and smoking ratio between the MeS group and the control group (*P* > 0.05 for all). Hypertension, hyperlipidemia, family history for coronary artery disease, BMI, and systolic and diastolic blood pressures were statistically significantly higher in the MeS group compared to the control group (*P* < 0.05 for all).

When the laboratory parameters and echocardiographic parameters were compared between the patient and the control groups, HDL, triglycerides, GGT, and fasting insulin levels were statistically significantly higher in the MeS group compared to the control group (*P* < 0.05 for all). When HOMA-IR levels were compared between the MeS and control groups for the insulin resistance, the HOMA-IR levels were statistically significantly higher in the MeS group compared to the control group (5.0 ± 2.2 and 1.4 ± 0.6, resp.; *P* < 0.001) (Table 1).

Also, the serum level of hs-CRP was statistically significantly higher in the MeS group compared to the control group (3.9 ± 0.5 mg/L and 0.7 ± 0.8 mg/L, resp.; *P* < 0.001) (Table 1).

**Figure 1:**
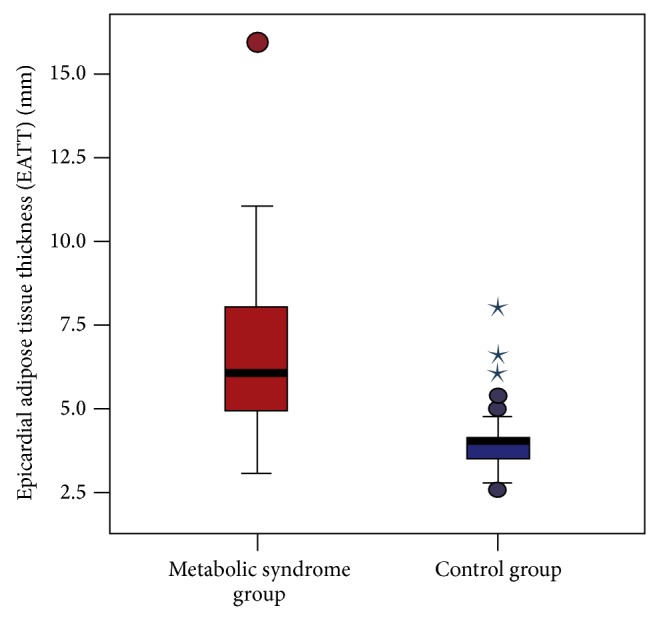
Comparison of EATT between patients with metabolic syndrome and the control group (6.0 ± 2.0 mm and 4.0 ± 1.0 mm, resp.; *P* < 0.001).

When the MeS group was compared with the control group for the epicardial adipose tissue thickness (EATT), EATT was statistically significantly higher in the control group (6.0 ± 2.0 mm and 4.0 ± 1.0 mm, resp.; *P* < 0.001) (Figure 1).

**Figure 2:**
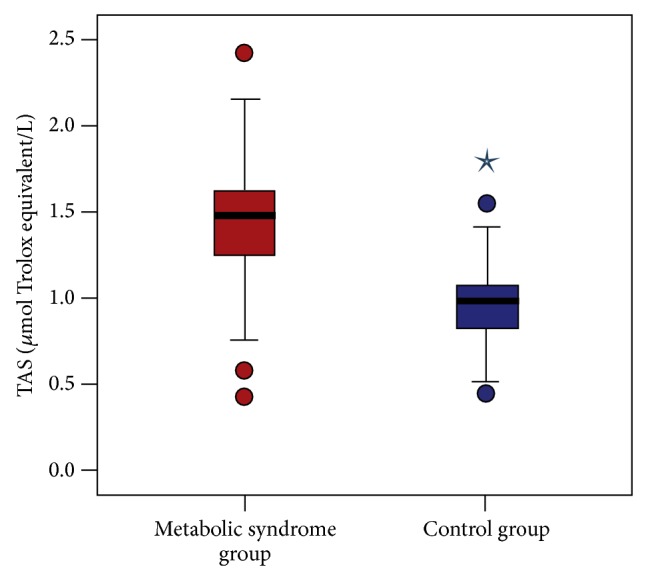
Comparison of serum TAS levels of patients with metabolic syndrome and the control group (1.4 ± 0.4 *μ*mol Trolox equivalent/L and 1.0 ± 0.2 *μ*mol Trolox equivalent/L, resp.; *P* < 0.001).

**Figure 3:**
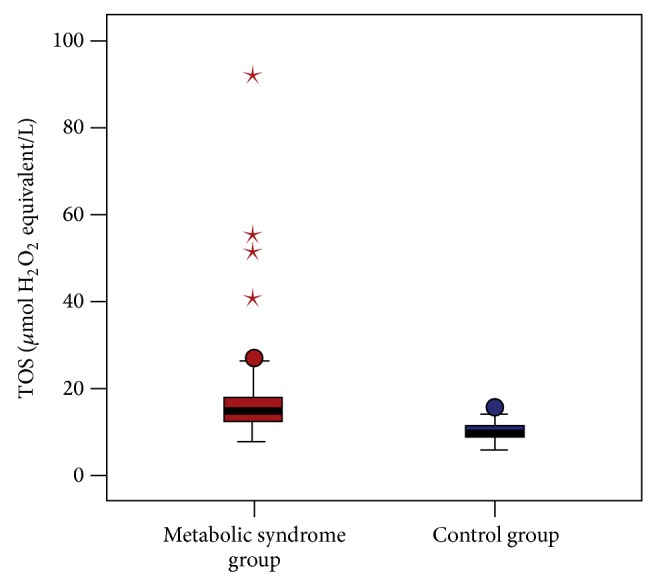
Comparison of serum TOS levels of patients with metabolic syndrome and the control group (17.3 ± 10.6 *μ*mol H_2_O_2_ equivalent/L and 10.4 ± 2.0 *μ*mol H_2_O_2_ equivalent/L, resp.; *P* < 0.001).

When the oxidative stress parameters were compared between the MeS and control groups, total oxidant status (TOS) was statistically significantly higher in the MeS group compared to the control group (17.3 ± 10.6 *μ*mol H_2_O_2_ equivalent/L and 10.4 ± 2.0 *μ*mol H_2_O_2_ equivalent/L, resp.; *P* < 0.001) (Figure 2). Additionally, the level of total antioxidant status (TAS) was statistically significantly higher in the MeS group compared to the control group (1.4 ± 0.4 *μ*mol Trolox equivalent/L and 1.0 ± 0.2 *μ*mol Trolox equivalent/L, resp.; *P* < 0.001). There was no statistically significant difference in oxidative stress index (OSI) between the two groups (13.3 ± 8.5 arbitrary unit and 11.7 ± 4.0 arbitrary unit, resp.; *P* = 0.843) (Figure 3).

When the MeS and control groups were compared for paraoxanase-1 (PON-1) and arylesterase, the serum levels of PON-1 and arylesterase were statistically significantly lower in the MeS group compared to the control group (112.4 ± 41.8 U/L and 214.2 ± 21.4 U/L; *P* < 0.001; 134.6 ± 40.6 U/L and 210.8 ± 39.5 U/L, resp.; *P* < 0.001) (Table 1).

In the correlation analysis between EATT and BMI, HOMA-IR, hs-CRP, TAS, TOS, OSI, paraoxonase-1, and arylesterase, EATT was positively correlated with BMI, HOMA-IR, hs-CRP, TAS, and TOS with statistical significance (*r* = 0.575, *r* = 0.567, *r* = 0.666, *r* = 0.308, and *r* = 0.339, resp.; *P* < 0.001 for all) (Table 2). Furthermore, a statistically significantly negative correlation was identified between EATT and paraoxonase-1 and arylesterase (*r* = −0.503, *P* < 0.001; *r* = −0.314, *P* < 0.001, resp.) (Table 2). No correlation was found between EATT and OSI (*r* = −0.041, *P* < 0.587) (Table 2).

In the single-variable linear regression analysis with EATT as the dependent variable and other parameters as independent variables, a significant correlation was found between EATT and BMI, HOMA-IR, hs-CRP, TAS, paraoxonase-1, and arylesterase (*P* < 0.01 for all) (Table 3). Furthermore, in the multivariable linear regression model with EATT as the dependent variable and other parameters as independent variables, a significant correlation was found between EATT and BMI and hs-CRP and TAS (*P* < 0.01 for all) (Table 3).

### 3.1. The Predictive Values of Epicardial Adipose Tissue Thickness and Oxidative Stress Parameters for the Diagnosis of Metabolic Syndrome

**Figure 4:**
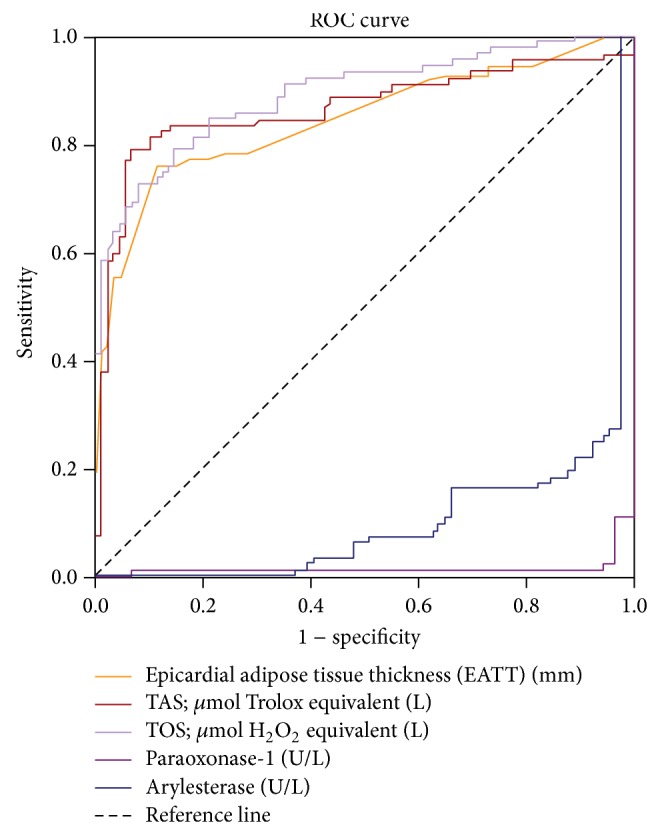
ROC curve of EATT, TAS, TOS, paraoxonase-1, and arylesterase for the detection of metabolic syndrome.

In the receiver operating characteristics (ROC) curve for EATT, EATT prediction values > 4.7 mm were associated with the diagnosis of MeS with a sensitivity of 76.1%, a specificity of 88.8%, a positive predictive value of 87.5%, and a negative predictive value of 78.2% (area under the curve: 0.82; 95% confidence interval: 0.76–0.89; *P* < 0.001) (Figure 4).

In the ROC curve for TAS, TAS prediction values > 1.2 *μ*mol Trolox equivalent/L were associated with the diagnosis of MeS with a sensitivity of 79.3%, a specificity of 92.1%, a positive predictive value of 91.3%, and a negative predictive value of 81.2% (area under the curve: 0.86; 95% confidence interval: 0.80–0.92; *P* < 0.001) (Figure 4). Similarly, in the ROC curve for TOS, TOS prediction values > 12.6 *μ*mol H_2_O_2_ equivalent/L were associated with the diagnosis of MeS with a sensitivity of 79.3%, a specificity of 85.4%, a positive predictive value of 84.9%, and a negative predictive value of 80% (area under the curve: 0.82; 95% confidence interval: 0.76–0.89; *P* < 0.001) (Figure 4).

In the ROC curve for PON-1, PON-1 prediction values < 180 U/L were associated with the diagnosis of metabolic syndrome with a sensitivity of 97.8%, a specificity of 95.5%, (area under the curve: 0.97; 95% confidence interval: 0.91–1.00; *P* < 0.001) (Figure 4). Similarly, in the ROC curve for arylesterase, ARE prediction values < 170 U/L were associated with the diagnosis of metabolic syndrome with a sensitivity of 72.8%, a specificity of 97.8%, (AUC: 0.85; 95% confidence interval: 0.79–0.91; *P* < 0.001).

## 4. Discussion

As a result of the present study, the epicardial adipose tissue thickness was found to have increased in patients with MeS, consistent with the literature. Additionally, the levels of TOS and TAS were also higher in MeS patients compared to the control group. Another interesting finding was the significantly reduced activity of paraoxonase-1 and arylesterase from the antioxidant enzymes in the MeS patients. To the best of the knowledge of the researchers, this is the first study in the literature that evaluates the epicardial adipose tissue thickness alongside the comprehensive oxidative stress parameters in the patient population with MeS.

Metabolic syndrome occurs when a number of risk factors come together, accompanied by a higher cardiometabolic risk. Visceral obesity has an important role in the development of MeS. The positive correlation of EATT and HOMA-IR with BMI in MeS patients, which was found in the present study, supports the hypothesis that epicardial adipose tissue is an important component of the visceral adipose tissue. Previous studies have demonstrated that epicardial adipose tissue acts as an active endocrine organ by producing many proinflammatory cytokines and bioactive adipokines [13]. Leptin and resistin, which are associated with a high cardiovascular risk, were found at a higher concentration in the epicardial adipose tissue [13]. On the other hand, the expression of adiponectin, which is an anti-inflammatory cytokine increasing the insulin sensitivity, was reduced in the epicardial adipose tissue in the patients with coronary artery disease [19]. Moreover, another important feature of epicardial adipose tissue is to play a role in the development of inflammation, which has a major part in the pathogenesis of DM, coronary artery disease, and MeS, in particular. In a study by Mazurek et al., the expression of IL-1, IL-6, IL-6sR, and TNF-alpha in the epicardial adipose tissue was greater compared to that in the subcutaneous adipose tissue in the patients with documented coronary artery disease [20]. In other words, epicardial adipose tissue contributes to the pathogenesis of MeS, by producing proinflammatory cytokines. In the present study, the level of hs-CRP, the indicator of a low-grade chronic inflammation, was higher compared to that in the control group. Additionally, a positive correlation was established between EATT, which was higher in the MeS patients, and hs-CRP, which is considered to be another important finding. In other words, visceral adipose tissue, such as epicardial adipose tissue, may directly contribute to the development of MeS due to its local and systemic proinflammatory effect.

Another important finding of the present study is that the levels of total oxidant status (TOS) and total antioxidative capacity (TAS) were higher in MeS patients. Today, the increased oxidative stress has a critical role in the pathogenesis of many cardiometabolic diseases. The impaired balance between the prooxidant system and the antioxidant system in favor of the prooxidant system results in low-grade inflammation, endothelial dysfunction, and insulin resistance, thereby coronary artery disease in MeS patients [8]. An experimental study conducted on pigs induced with a high-calorie diet demonstrated that NO bioavailability was reduced and thereby endothelium-dependent vasodilation was impaired [21]. This result is explained by obesity leading to increased oxidative stress and causing endothelial dysfunction [7, 21]. Furthermore, Furukawa et al. demonstrated that lipid peroxidation, which is an indicator of increased oxidative stress, is associated with BMI and waist circumference in MeS patients [22]. Parallel to this, another study on the MeS patients demonstrated that the most important determinant of urinary 8-epi-PGF2a levels, which are a significant indicator of increased oxidative stress, is visceral adipose tissue [23]. All these data suggest that the visceral adipose tissue also accompanies an increase in oxidative stress. Higher EATT values and the positive correlation between EATT and TOS that were found in the present study suggest that epicardial adipose tissue, which is an important component of visceral adipose tissue, also leads to increased oxidative stress, thereby playing a role in the development of MeS. As a matter of fact, one study conducted to support this opinion demonstrated that the epicardial adipose tissue involves more oxidative stress elements compared to the subcutaneous adipose tissue [10]. However, one of the interesting results was that the TAS level was higher in MeS patients in the present study. However, the TAS level would have been expected to be lower in the MeS group. This, of course, seems to be a paradoxical result. In fact, a few studies in the literature have found lower TAS levels in MeS patients when compared to a control group [24, 25]. This paradoxical result that was obtained in the present study may have resulted from the increased antioxidant activity as a compensatory response to increased oxidative stress. Additionally, a recent study by Torun et al. on MeS patients found higher TAS levels in the MeS group, which is parallel to the present study [26]. There are also some studies with no difference in TAS levels between MeS patients and the control group [27]. On the other hand, the finding of a positive correlation between TAS and EATT in the present study suggests that epicardial adipose tissue has an important role, not only in increased oxidative stress but also in the compensatory response to the increased oxidative stress. Moreover, another reason for higher TAS levels in the present study may be due to the patients being composed of isolated MeS group and having a relatively younger age. This is due to MeS patients with DM and coronary artery disease not being included in the present study. In conclusion, the association of TAS levels with MeS seems to be of conflict and new data are required in order to explicitly clarify this matter.

Another important finding of the present study was the apparently low activity of paraoxonase-1 (PON-1) and arylesterase (ARE) in MeS patients. As is known, PON-1 is a glycoprotein, which is mainly synthesized by the liver and associated with the high-density lipoprotein [27]. It is known that this enzyme protects low-density lipoproteins from oxidation by ensuring the hydrolysis of lipid peroxides [27]. In other words, PON-1 is an antioxidant enzyme. Serum ARE activity correctly reflects PON-1 activity and is not affected by the PON-1 polymorphism [27]. As in the present study, Hashemi et al. found low PON-1 and ARE activity in patients with MeS [27]. Nevertheless, Tabur et al. did not find low PON-1 activity in nondiabetic patients with MeS, which was contrary to the present study, and they observed no difference in PON-1 between the control group and MeS patients [28]. However, PON-1 produced a high sensitivity and specificity for the prediction of MeS patients in the ROC analysis in the present study. Another important result of the present study is the evident negative correlation of EATT with PON-1 and ARE. In other words, the serum PON-1 activity decreases as EATT increases in the patient group with MeS. A recent study by Ertem et al. did not establish a significant correlation between EATT and PON-1 in the individuals without any coronary artery disease [29]. However, they found a negative correlation, as in the present study, between EATT and PON-1 in the subgroup with EATT ≥ 7 mm [29]. The result of this subgroup analysis seems to be consistent with the present study. In other words, the antioxidant PON-1 activity decreases as EATT increases. The study by Jayakumari and Thejaseebai found low PON-1 activity in patients with a coronary artery disease [30] Therefore, PON-1 shows antiatherosclerotic properties. Although the present study excluded patients with documented coronary artery disease, the low levels of PON-1 may be an indicator of increased cardiovascular risk and a symptom of subclinical coronary artery disease in the patient group with MeS. In conclusion, there are conflicting results for PON-1 activity in the patient group with MeS. The present study is important, as it is the first in the literature, as far as the authors know, which evaluates the association between the PON-1 levels and epicardial adipose tissue in MeS.

Another finding of the present study is the strong positive correlation between EATT and HOMA-IR. In fact, this is an expected finding in the patient group with metabolic syndrome. This is because epicardial adipose tissue is a component of visceral adipose tissue and its increased amount in MeS patients contributes to increased insulin resistance. There are also some studies that have established a significant correlation between epicardial adipose tissue and insulin resistance, supporting the present study [31, 32].

The present study has some limitations. The main limitation is the low number of patients. However, the low number of patients is due to the exclusion of patients with diabetes and coronary artery disease. In other words, it is simply due to the inclusion of the patients with isolated MeS. Another important limitation is the measurement of the epicardial adipose tissue of the patients by echocardiography alone, even though there seems to be a correlation with other imaging techniques such as MR and CT. Additionally, a single measurement for oxidative stress parameters and the lack of long-term follow-up in these patients can be considered limitations in terms of evaluating the long-term results.

In conclusion, this is the first study in the literature, as far as the authors know, which simultaneously evaluates epicardial adipose tissue thickness and oxidative stress parameters, the levels of paraoxonase-1, arylesterase, hs-CRP, and insulin resistance in a patient population with MeS. Large-scale studies on this matter are required in order to better establish the correlation between the epicardial adipose tissue thickness and oxidative stress.

## Figures and Tables

**Table 1:** Comparison of personal information, clinical features, and laboratory parameters between metabolic syndrome patients and the control group.

Parameter	Metabolic syndrome *N* = 92		Control *N* = 88		*P* value
	Mean ± SD	Median, 1.Q–3.Q	Mean ± SD	Median, 1.Q–3.Q	
Age, years	39.9 ± 11.7	40, 18–73	39.0 ± 9.6	40, 18–64	0.532
Gender (male/female)	25/67	—	17/72	—	0.198
Smoking, *n*	33	—	27	—	0.429
Hypertension, *n*	56	—	4	—	<0.001
Hyperlipidemia, *n*	15	—	4	—	0.01
Family history, *n*	57	—	30	—	<0.001
Waist circumference, cm	111 ± 13.6	110, 85–165	86.6 ± 12.0	85, 64–125	<0.001
BMI, kg/m^2^	35.0 ± 7.0	34, 21.7–58.5	25.9 ± 4.5	25, 18.3–40.1	<0.001
Systolic blood pressure, mmHg	134.3 ± 21.9	130, 90–190	111.1 ± 13.1	110, 80–140	<0.001
Diastolic blood pressure, mmHg	83.9 ± 12.0	80, 50–100	70.9 ± 9.6	70, 50–90	<0.001
Glucose, mg/dL	96.9 ± 11.4	96, 64–121	85.3 ± 10.1	86, 61–116	<0.001
TCHOL, mg/dL	188.5 ± 38.2	188, 89–279	181.2 ± 40	171, 111–292	0.077
LDL, mg/dL	110.4 ± 29.8	110, 40–186	106.3 ± 33.9	101, 36–208	0.107
HDL, mg/dL	44.2 ± 11.0	44, 23–97	51.7 ± 13.6	48, 29–101	<0.001
TG, mg/dL	181.2 ± 87.1	169, 48–525	108.3 ± 66.8	85, 20–403	<0.001
GGT, U/L	28.1 ± 18.8	22, 10–111	22.4 ± 27.9	16, 6–234	<0.001
Urea, mg/dL	27.0 ± 10.0	26, 12–88	24.6 ± 6.7	23, 12–45	0.510
Creatinine, mg/dL	0.7 ± 0.2	1, 0.4–1.3	0.7 ± 0.4	1, 0.3–3.8	0.599
Hemoglobin, g/dL	13.0 ± 1.7	13, 8.4–17.8	12.8 ± 1.5	13, 8.7–17	0.320
Insulin, mIU/mL	21.4 ± 9.2	18, 9.7–63.4	17.4 ± 98.5	7, 2.0–936	<0.001
hs-CRP, mg/L	3.9 ± 0.5	4, 3.3–6.7	0.7 ± 0.8	1, 0.3–5.9	<0.001
HOMA-IR	5.0 ± 2.2	4, 2.6–12.6	1.4 ± 0.6	1, 0.3–3.3	<0.001
TOS, *μ*mol H_2_O_2_ equivalent/L	17.3 ± 10.6	15, 7.9–91.3	10.4 ± 2.0	10, 6.1–15.9	<0.001
TAS, *μ*mol Trolox equivalent/L	1.4 ± 0.4	1, 0.4–2.4	1.0 ± 0.2	1, 0.4–1.8	<0.001
OSI, arbitrary unit	13.3 ± 8.5	10, 6.0–56.7	11.7 ± 4.0	11, 5.1–24.8	0.843
Paraoxonase-1, U/L	112.4 ± 41.8	107, 50.7–249.6	214.2 ± 21.4	212, 162.8–275.1	<0.001
Arylesterase, U/L	134.6 ± 40.6	115, 80.9–220	210.8 ± 39.5	200, 52.4–293.1	<0.001
EATT, mm	6.0 ± 2.0	1, 3–16	4.0 ± 1.0	0, 3–8	<0.001

BMI, body mass index; TCHOL, total cholesterol; LDL, low-density lipoprotein; HDL, high-density lipoprotein; TG, triglyceride; GGT, gamma-glutamyl transferase; hsCRP, high sensitive C-reactive protein; HOMA-IR, homeostatic model assessment-insulin resistance; TAS, total antioxidant status; TOS, total oxidant status; OSI, oxidative stress index; EATT, epicardial adipose tissue thickness.

**Table 2:** Results of the correlation analysis between EATT and other parameters.

Parameter		BMI	HOMA-IR	hs-CRP	TAS	TOS	OSI	Paraoxonase-1	Arylesterase
EATT	*r*	0.575	0.567	0.666	0.308	0.339	−0.041	−0.503	−0.314
	*P*	0.000	0.000	0.000	0.000	0.000	0.587	0.000	0.000

EATT, epicardial adipose tissue thickness; BMI, body mass index; HOMA-IR, homeostatic model assessment-insulin resistance; hs-CRP, high sensitive C-reactive protein; TAS, total antioxidant status; TOS, total oxidant status; OSI, oxidative stress index.

**Table 3:** Results of univariate and multivariate linear regression analysis to evaluate the effect of independent variables on EATT.

Univariate model	*B*	SE	Beta	*t*	*P*
BMI	0.01	0.00	0.47	7.07	0.000
HOMA-IR	0.04	0.01	0.48	7.36	0.000
hs-CRP	0.08	0.01	0.63	10.82	0.000
TAS	0.13	0.04	0.23	3.17	0.002
TOS	0.00	0.00	0.15	1.97	0.05
OSI	0.00	0.00	0.06	0.75	0.45
Paraoxonase-1	0.00	0.00	−0.43	−6.42	0.000
Arylesterase	0.00	0.00	−0.31	−4.32	0.000

Multivariate model	*B*	SE	Beta	*t*	*P*

BMI	0.01	0.00	0.18	2.69	0.008
hs-CRP	0.08	0.01	0.64	8.36	0.000
TAS	−0.11	0.04	−0.20	−2.96	0.003

*B*, unstandardized coefficients; SE, standard error; beta, standardized coefficients; EATT, epicardial adipose tissue thickness; BMI, body mass index; HOMA-IR, homeostatic model assessment-insulin resistance; hs-CRP, high sensitive C-reactive protein; TAS, total antioxidant status; TOS, total oxidant status; OSI, oxidative stress index.
